# Identification of novel mutations in glucocerebrosidase (GBA) gene in Indian patients with gaucher disease (GD)

**DOI:** 10.1186/1755-8166-7-S1-P52

**Published:** 2014-01-21

**Authors:** Chitra Ankleshwaria, Jayesh Sheth, Mehul Mistri, Ashish Bavdekar, Sheela Nampoothiri, Sarita Gupta, Frenny Sheth

**Affiliations:** 1 Institute of Human Genetics, FRIGE House, Jodhpur Gam Road, Satellite, Ahmedabad, India; 2 KEM Hospital, 489, Rasta peth, Sardar Mudliar Road, Pune, India; 3 Amrita Institute of Medical Sciences and Research Centre, AIMS Ponekkara, Kochi, India; 4 Department of Biochemistry, The MS University, Baroda, India

**Keywords:** Gaucher disease (GD), Glucocerebrosidase (*GBA*) gene, India, Mutation analysis

## Background

Gaucher disease (GD) is the most common glycolipid storage disorder due to inherited deficiency of lysosomal enzyme acid β-glucosidase (glucocerebrosidase, E.C.3.2.1.45) occurring due to mutations in the GBA gene. More than 300 mutations have been reported with higher frequency of most common mutant allele N370S (c.1226A>G), Leu29AlafsX18 (c.84dupG), *L444P* (c.1448T>C), IVS2+1G > A (c.115+1G>A) in Jewish population. The objective of the investigation was to identify mutations in Indian patients with GD and to understand genotype/phenotype correlation.

## Materials and methods

Our study comprises of forty five patients with GD and we have reported two novel mutations observed in two of the patients. Genomic DNA was extracted from whole blood by salting-out method and was screened for the common N370S (c.1226A>G), *L444P* (c.1448T>C), R463C (c.1504C>T), and IVS2 (+1) G>A (c.115+1G>A) mutations by RFLP PCR. Bidirectional sequencing was carried out using automated sequencer in absence of above common mutations. In silico analysis was carried out using Polyphen2, SIFT and Mutation t@sting softwares.

## Results

Our study has identified two novel missense mutations G289A (c.866G>A) in homozygous state in Exon-7 and I466S (c.1397T>G) in heterozygous state in Exon-10 in two patients respectively. Experimental program Polyphen 2 showed G289A (c.866G>A) and I466S (c.1397T>G) as probably damaging with a score of 0.963 (sensitivity: 0.78, specificity: 0.95) and 1.000 (sensitivity: 0.00, specificity: 1.00) respectively. SIFT/PROVEAN Human program showed G289A (c.866G>A) and I466S (c.1397T>G) mutation as deleterious with score of -3.233 and -5.045 respectively and Mutation testing showed G289A (c.866G>A) as disease causing mutation and I466S (c.1397T>G) mutation as polymorphism.

## Conclusion

In this study, we have observed two novel GD mutations G289A (c.866G>A) and I466S (c.1397T>G) and effect of this genotype was studied using protein modeling. Identification of the genotype helps in predicting phenotypic expression, therapeutic response, and carrier screening for genetic counselling.

